# The Peripheral Nervous System in Amyotrophic Lateral Sclerosis: Opportunities for Translational Research

**DOI:** 10.3389/fnins.2019.00601

**Published:** 2019-06-25

**Authors:** Francesco Gentile, Stefania Scarlino, Yuri Matteo Falzone, Christian Lunetta, Lucio Tremolizzo, Angelo Quattrini, Nilo Riva

**Affiliations:** ^1^Experimental Neuropathology Unit, Division of Neuroscience, Institute of Experimental Neurology – San Raffaele Scientific Institute, Milan, Italy; ^2^Department of Neurology, San Raffaele Scientific Institute, Milan, Italy; ^3^NeuroMuscular Omnicentre, Serena Onlus Foundation, Milan, Italy; ^4^Neurology Unit, ALS Clinic, San Gerardo Hospital, University of Milano-Bicocca, Monza, Italy

**Keywords:** motor neuron disease, lower motor neuron syndrome, nerve, neuropathy, CMT, distal SMA, hereditary neuropathy, genetics

## Abstract

Although amyotrophic lateral sclerosis (ALS) has been considered as a disorder of the motor neuron (MN) cell body, recent evidences show the non-cell-autonomous pathogenic nature of the disease. Axonal degeneration, loss of peripheral axons and destruction of nerve terminals are early events in the disease pathogenic cascade, anticipating MN degeneration, and the onset of clinical symptoms. Therefore, although ALS and peripheral axonal neuropathies should be differentiated in clinical practice, they also share damage to common molecular pathways, including axonal transport, RNA metabolism and proteostasis. Thus, an extensive evaluation of the molecular events occurring in the peripheral nervous system (PNS) could be fundamental to understand the pathogenic mechanisms of ALS, favoring the discovery of potential disease biomarkers, and new therapeutic targets.

## Introduction

Amyotrophic lateral sclerosis (ALS) is the most common and severe form of motor neuron disease (MND), an heterogeneous group of disorders defined by prominent motor neuron (MN) degeneration (Saberi et al., 2015; Riva et al., 2016). Recent evidence has challenged such traditional view of selective neuronal loss demonstrating widespread extra-motor involvement in ALS and implying that neuronal populations other than MNs may also be affected (Geser et al., 2008; Brettschneider et al., 2013; McCluskey et al., 2014). Furthermore, an expanded clinical spectrum has now been recognized, as overlapping phenotypes with other neuromuscular disorders (Sabatelli et al., 2016), including peripheral neuropathies, have been described (Rajabally and Jacob, 2008; Bhatt et al., 2009; Sawa et al., 2012). Although in clinical practice MNDs should be differentiated from other peripheral nervous system (PNS) disorders, sensory and autonomic neurons in the dorsal root ganglia (DRG) and lower MNs in the ventral horns share important challenges, as proper stability and functioning of their long projections throughout the body requires a protective environment and efficient communication between the central nervous system (CNS) and the outermost areas of these cells. Nonetheless, differences in morphology and function may confer different patterns of resistance or vulnerability to specific stressors.

The aim of this review is to dissect PNS involvement in ALS, analyzing the evidence from clinical and pathological data to genetics in order to provide novel pathophysiological insights and potential implication for therapeutic strategies.

## Diagnostic Challenges in ALS and ALS-Mimics

**FIGURE 1:**
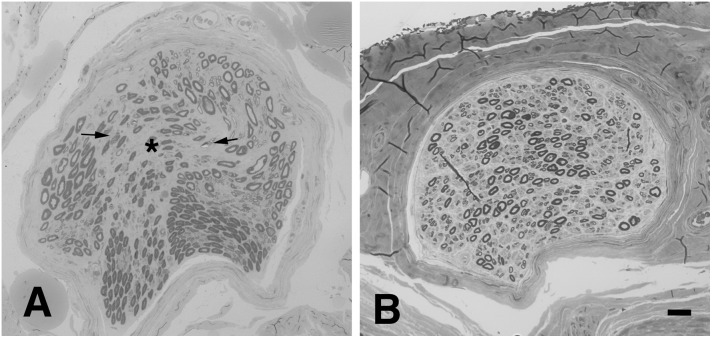
Representative neuropathological cases. **(A)** Transverse semi-thin sections of motor nerve biopsy (obturator nerve), and sural sensory nerve biopsy **(B)** from a patients with MND. **(A)** Focal loss of myelinated nerve fibers is evident (asterisk) in the endoneurium together with signs of acute axonal degeneration (myelin ovoids, arrows); no signs of regeneration are observed. **(B)** In sural sensory nerve biopsies a diffuse mild reduction of large nerve fibers is observed. Bar: 20 μm.

In the absence of pathognomonic diagnostic biomarkers, ALS diagnosis relies on clinical findings suggestive of selective upper, and lower MN (LMN) lesion and the exclusion of alternative causes (Ludolph et al., 2015). The wide clinical heterogeneity of the disease led to the development of several phenotypic classifications, mainly dependent on the distribution of UMN and LMN lesions. In patients presenting with isolated signs of LMN involvement (LMN Syndrome – LMNS), the differential diagnosis may be more challenging since the primary disease target may be the cell soma, the axon and its myelin, the neuromuscular junction (NMJ) or the muscle (Riva et al., 2011b; Garg et al., 2017; Muller et al., 2018). Such disorders not only share the motor unit as a common target of damage, but may be potentially underpinned by common pathogenic mechanisms. Within this context, ALS, with special reference to its restricted LMN phenotypes, such as flair legs and flail arm syndromes, shows high overlap in terms of clinical presentation, targets and mechanisms of damage with hereditary axonal neuropathies, such as axonal Charcot-Marie-Tooth neuropathy (CMT2) and distal hereditary motor neuropathy (dHMN) (Fischer and Glass, 2007). Besides the different rate of progression and spreading, distinction between these disorders could be difficult especially in the early phase, as clinical and neurophysiologic studies may be strikingly similar and may not able to discern the primary lesion site. Nonetheless, a recent study supported the value of the electroneurographic split hand index has recently been suggested for distinguishing ALS from mimic disorders (Menon et al., 2014). Moreover, pes cavus, traditionally considered a distinguishing clinical sign of CMT, has also been anecdotally reported in ALS patients (Ceroni et al., 2001), and is estimated to be as high as 2% our case series (data not published). Notably, the percentage of misdiagnosis in degenerative LMNS has been reported to be as high as 19%; moreover, up to 10% of patients initially diagnosed as ALS are ultimately re-diagnosed as having another disease, including peripheral neuropathy (Davenport et al., 1996; Traynor et al., 2000; Visser et al., 2002). Therefore, in some cases, MND diagnosis remains uncertain, and only follow up can lead to a satisfying level of diagnostic confidence. Of note, we previously showed that biopsy of obturator nerve may be useful in the distinction between ALS and motor neuropathies (Corbo et al., 1997; Riva et al., 2011b). Signs of acute axonal damage, focal/multifocal fiber loss and absent or scarce axonal regeneration are consistently observed in motor nerve biopsies from patients with ALS (Figure 1A), while neuropathies show a higher regeneration capacity and may present signs of myelin damage, inflammation, or pathologic deposits (Benedetti et al., 2010).

## The PNS as a Converging Point in ALS

### Evidence of Peripheral Nervous System Involvement in Human ALS

Although the circumstances leading to MN damage in ALS are still unknown, different mechanisms have been proposed to explain disease onset and spread. The dying-forward hypothesis states that the primary site of damage resides in the cell soma and subsequently spreads to the peripheral compartments. In the recent years, the dying-back pattern of degeneration has obtained a large attention in the context of ALS pathophysiology (Fischer et al., 2004), suggesting that ALS is a distal axonopathy, a pattern typically seen in peripheral neuropathies with a distal to proximal gradient of damage (England and Asbury, 2004). Therefore, considering that both motor and sensory nerves share mechanisms of axonal degeneration, it may be plausible that in ALS signs of PNS dysfunction may also extend to the sensory system. Notably, other neurodegenerative diseases such as Parkinson’s disease and Alzheimer’s disease show evidence of distal axonopathy (Arendt, 2009; Chung et al., 2009).

While diagnostic assessment in ALS relays on the assumption that non-motor components of the PNS are usually spared, increasing amount of evidence suggests the presence of sensory and autonomic dysfunction in ALS. Sensory symptoms are reported in about 2–30% of cases (Gubbay et al., 1985; Hammad et al., 2007). Numbness, tingling and pain are the most common symptoms reported. Decreased vibration sense and abnormal pain and temperature thresholds are rarely observed during standard examination, however quantitative sensory testing shows abnormal findings in about 10% of patients, rising to almost half of the cases in advanced stages of the disease (Truini et al., 2015; Isak et al., 2017).

Autonomic dysfunction has been reported in 5–30% of cases, while quantitative autonomic testing detected abnormal sudomotor and cardiovagal response in up to 50% of patients (McCluskey et al., 2014; Piccione et al., 2015). Abnormalities in either sensory nerve amplitudes or nerve conduction velocity may be apparent in about 20% of cases (Mondelli et al., 1993; Pugdahl et al., 2008), with higher frequencies reported in patients with longer disease duration (Pugdahl et al., 2007; Isak et al., 2016).

Pathologic studies of the sural nerve in ALS have demonstrated a mild reduction of the number of large nerve fibers (Figure 1B) in most of the examined cases (Bradley et al., 1983; Hammad et al., 2007; Devigili et al., 2011; Luigetti et al., 2012). Clustering of mitochondria, dilation of small vesicles and accumulation of neurofilaments (NF) have also been reported (Dyck et al., 1975; Di Trapani et al., 1987; Heads et al., 1991). Skin biopsy shows in about 80% of ALS patients a reduction of intraepidermal nerve fiber density (Weis et al., 2011; Truini et al., 2015; Dalla Bella et al., 2016; Nolano et al., 2017) and focal axonal swellings, suggesting axonal degeneration (Isak et al., 2017). Interestingly, small nerve fibers in the cornea are also reduced in ALS patients, correlating with bulbar disability scores (Ferrari et al., 2014). Finally, a significant neuronal loss has been observed in DRG of ALS patients, with preferential involvement of large-sized cells (Kawamura et al., 1981). Notably, evidence of pTDP-43 pathology has been shown in Clarke’s column, intermediolateral nucleus and dorsal root ganglia in a subset of cases, proving that these structures are also affected (Nishihira et al., 2008; Brettschneider et al., 2014).

### The Role of Genetics in ALS: At the Borders of the Disease Spectrum

The knowledge about ALS genetics has had a relevant acceleration in the last decade, with more than 30 genes identified as pathogenic and more than 100 as ALS-related (Riva et al., 2016). The first gene identified as a cause of ALS was *SOD1* in 1993 (Rosen et al., 1993) and together with *TARDBP* (Sreedharan et al., 2008), *FUS* (Kwiatkowski et al., 2009), and chromosome 9 open reading frame 72 (*C9orf72*) (DeJesus-Hernandez et al., 2011; Renton et al., 2011), represent the most common ALS-related genes, covering up to 70% of familial and 10% of sporadic cases (Riva et al., 2016; Chia et al., 2018). ALS-key genes have been associated with almost all clinical ALS phenotypes, although patients with SOD1, and FUS mutations may show preferential LMN involvement (Waibel et al., 2013; Picher-Martel et al., 2016).

The frequency of gene variants in cases with extra-motor involvement is significantly higher compared to the pure ALS phenotype, and has been associated with poorer survival (McCluskey et al., 2014). Prominent sensory involvement had been described in rare mutated cases, with *SOD1* and *TARDBP* genes being reported most frequently (Camdessanche et al., 2011).

Many of the genes involved in ALS are associated also with other neurological disorders sharing common targets of neurodegeneration, such as hereditary spastic paraplegia (HSP), axonal CMT neuropathy and dHMN. Among the genes involved in dHMN and axonal CMT there are Berardinelli-Seip Congenital Lipodystrophy 2 (*BSCL2*), neurofilament light (*NEFL*), transient receptor potential cation channel subfamily V member 4 (*TRPV4*), heat shock protein family B1, B3, and B8 (*HSPB1*, *HSPB3*, and *HSPB8)* (Martini et al., 2000; Rossor et al., 2012). Despite the discrepancy between the major genes mutated in hereditary neuropathies and ALS, each of them exerts pleiotropic functions in neuronal homeostasis, including RNA metabolism, protein quality control, axonal transport, stress response. Therefore, generation of a spectrum of clinical phenotypes from alteration in master genes, broadly involved in key neuronal metabolic pathways, could be expected (Table 1).

**Table 1:** Key genes associated with ALS, hereditary neuropathy, and overlapping phenotypes.

Gene	Gene name	Chromosome	Disease^∗^	Inheritance	References
**ALS genes**		
C9orf72	Chromosome 9 open reading frame 72	9p21	FTD and/or ALS 1	AD	DeJesus-Hernandez et al., 2011; Renton et al., 2011
FUS	FUS RNA Binding Protein	16p11	ALS6 with or without FTD	AD AR	Vance et al., 2009
OPTN	Optineurin	10p13	Open angle glaucoma – ALS12	AD AR	Rezaie et al., 2002 (Glaucoma); Maruyama et al., 2010 (ALS)
PFN1	Profilin 1	17p13	ALS18	AD	Wu et al., 2012
SOD1	Superoxide dismutase 1	21q22	ALS1	AD AR	Rosen et al., 1993
SQSTM1	Sequestosome 1	5q35	Paget disease of bone 3 – FTD and/or ALS3	AD	Laurin et al., 2002 (Paget Disease); Fecto et al., 2011 (ALS)
TARDBP	TAR DNA binding protein	1p36	ALS 10 with or without FTD	AD	Gitcho et al., 2008; Kabashi et al., 2008
UBQLN2	Ubiquilin 2	Xp11.21	ALS15 with or without FTD	X-linked; AD	Deng et al., 2011
**Overlap genes**		
DCTN1	Dynactin subunit 1	2p13	dHMNVIIB – Perry syndrome – ALS	AD/AR Risk factor (ALS)	Puls et al., 2003 (dHMN); Munch et al., 2005 (ALS); Farrer et al., 2009 (Perry syndrome)
DYNC1H1	Dynein cytoplasmic 1 heavy chain 1	14q32	CMT2O – dSMA1 – ALS	AD	Munch et al., 2004 (ALS); Weedon et al., 2011 (CMT); Harms et al., 2012 (dSMA)
FIG4	FIG4 phosphoinositide 5-phosphatase	6q21	CMT4J – ALS11 – Yunis-Varon syndrome	AR – AD	Chow et al., 2007, 2009 (CMT, ALS); Campeau et al., 2013 (Yunis-Varon)
GARS	Glycyl-tRNA synthetase	7p15	CMT2D – dHMNVA – ALS	AD	Antonellis et al., 2003 (CMT – dSMAV); Kruger et al., 2016 (ALS)
KIF5A	Kinesin family member 5A	12q13	CMT2 – SPG10 – ALS25	AD	Reid et al., 2002; Crimella et al., 2012 (HSP – CMT2); Nicolas et al., 2018 (ALS)
MFN2	Mitofusin 2	1p36	CMT2A2A – CMT2A2B – dHMNVIA – ALS like	AD AR	Zuchner et al., 2004a (CMT2A2A); MMarchesi et al., 2011 (ALS); Polke et al., 2011 (CMT2A2B)
NEFH	Neurofilament heavy	22q12	ALS – CMT2CC	AD – AR	Figlewicz et al., 1994 (ALS); Rebelo et al., 2016 (CMT)
PLEKHG5	Pleckstrin homology and RhoGEF domain containing G5	1p36	CMTC; SMA – SMA distal4 – ALS	AR	Maystadt et al., 2007 (SMA); Azzedine et al., 2013; Kim et al., 2013 (CMT); Ozoguz et al., 2015 (ALS)
SETX	Senataxin	9q34	dHMN with pyramidal signs – ALS4 juvenile – spinocerebellar ataxia 1	AD – AR	De Jonghe et al., 2002 (dHMN); Chen et al., 2004 (ALS); Moreira et al., 2004 (spinocerebellar ataxia)
SIGMAR1	Sigma non-opioid intracellular receptor 1	9p13	ALS16 juvenile – SMA distal 2	AR	Al-Saif et al., 2011 (ALS); Li X. et al., 2015 (dSMA2)
SPAST	Spastin	2p22	SPG4 – ALS juvenile	AD	Fonknechten et al., 2000 (HSP); Meyer et al., 2005 (ALS)
SPG11	Spatacsin vesicle trafficking associated	15q21	SPG11 – ALS5 juvenile – CMT2X	AR	Stevanin et al., 2007 (SPG); Orlacchio et al., 2010 (ALS); Montecchiani et al., 2016 (CMT)
VCP	Valosin containing protein	9p13	IBMPFD- ALS14 with or without FTD – CMT2Y	AD	Watts et al., 2004 (IBMPFD); Johnson et al., 2010 (ALS); Gonzalez et al., 2014 (CMT)
**CMT/dHMN genes**		
BSCL2	BSCL2, seipin lipid droplet biogenesis associated	11q13	Lipodystrophy, congenital generalized, type 2 – dHMNVA – silver spastic paraplegia syndrome	AR – AD	Magré et al., 2001 (Lypodistrophy); Windpassinger et al., 2004 (dHMNVA, Silver syndrome)
HSPB1	Heat shock protein family B (small) member 1	7q11	CMT2F – dHMN IIB	AD	Evgrafov et al., 2004 (CMT dHMN)
HSPB3	Heat shock protein family B (small) member 3	5q11	dHMN IIC	AD	Kolb et al., 2010
HSPB8	Heat shock protein family B (small) member 8	12q24	dHMN IIA – CMT2L	AD	Irobi et al., 2004 (dHMN); Tang et al., 2005 (CMT)
NEFL	Neurofilament light	8q21	CMT2E – CMT1F – CMTG	AD AR	Mersiyanova et al., 2000 (CMT2E); Jordanova et al., 2003 (CMT1F); Zuchner et al., 2004b (CMT2G)
TRPV4	Transient receptor potential cation channel subfamily V member 4	2q24	HMSN IIC – SMA – scapuloperoneal SMA	AD	Auer-Grumbach et al., 2010


***^∗^**OMIM and report from literature. ALS, amyotrophic lateral sclerosis; FTD, frontotemporal dementia; CMT, charcot marie tooth; SMA, spinal muscular atrophy; SPG, spastic paraplegia; HMSN, hereditary motor and sensory neuropathy; Dhmn, distal hereditary motor neuropathy; UPS, ubiquitin proteasome system; ERAD, endoplasmic reticulum-associated protein degradation; IBMPFD, inclusion body myopathy paget disease frontotemporal dementia*.

Neurofilaments (NF) are composed of three subunits defined by their molecular weight and encoded by the NF light (*NEFL*), medium (*NEFM*), and heavy (*NEFH*) genes (Lieberburg et al., 1989). NFs are specifically expressed in neurons and are the most abundant cytoskeletal components of myelinated axons, regulating their caliber, growth, and conduction rate (Hoffman et al., 1987). Mutations in *NEFL* are known to cause both axonal and demyelinating forms of CMT with different phenotypes, including pyramidal signs (Mersiyanova et al., 2000; Jordanova et al., 2003; Rebelo et al., 2016; Jacquier et al., 2017). Mutations in NEFH gene are involved in the pathogenesis of sporadic ALS (Figlewicz et al., 1994; Al-Chalabi et al., 1999) but also in CMT (Jacquier et al., 2017; Nam et al., 2017).

The cellular abundance of PI(3,5)P2, a phosphoinositide involved in the control of vesicles trafficking, is regulated by a phosphoinositide 5-phosphatase encoded by the *FIG4* gene. CMT4J cases, clinically characterized by early onset and aggressive disease progression, have been associated with by an autosomal dominant pattern of transmission and by biallelic *FIG4* mutations (Nicholson et al., 2011). Notably, heterozygous autosomal dominant *FIG4* variants have been more recently associated with ALS and identified as ALS11 (Osmanovic et al., 2017).

The valosin containing protein (VCP) is member of the AAA ATPase family of proteins. This protein is ubiquitously expressed, and it is implicated in multiple cellular processes, such as cell survival (Vandermoere et al., 2006; Braun and Zischka, 2008), stress response and DNA and protein quality control (DeLaBarre et al., 2006; Ju et al., 2009; Weihl et al., 2009). Mutations in *VCP* have been described in patients with autosomal dominant inclusion body myopathy (IBM) associated with Paget disease and fronto-temporal dementia (FTD) (IBMPFD) (Watts et al., 2004), pure ALS patients (Johnson et al., 2010; Miller et al., 2012), and recently also CMT (Gonzalez et al., 2014; Jerath et al., 2015).

The exact function of senataxin (SETX) is unknown but it may be involved in RNA metabolism. Studies have shown a role in DNA transcription and repair (Suraweera et al., 2009; Skourti-Stathaki et al., 2011). Mutations in *STX* have been described in ataxia-ocular apraxia 2 (AOA2) (Moreira et al., 2004; Duquette et al., 2005; Fogel and Perlman, 2006;Arning et al., 2008; Airoldi et al., 2010; Fogel et al., 2014), autosomal dominant juvenile ALS (Chen et al., 2004; Zhao et al., 2009; Avemaria et al., 2011; Arning et al., 2013; Tripolszki et al., 2017), and in dHMN with pyramidal features (De Jonghe et al., 2002).

The Spastic Paraplegia 11 gene (*SPG11*) encodes the spatacsin protein, selectively expressed in neuron with a role in axonal growth, transport, and cytoskeletal stability (Perez-Branguli et al., 2014). *SPG11* variants were first described in patients with autosomal recessive spastic paraplegia 11 with thin corpus callosum (Hehr et al., 2007). Then, descriptions in juvenile ALS (ALS5) and in CMT2X were also reported (Orlacchio et al., 2010; Daoud et al., 2012; Iskender et al., 2015; Montecchiani et al., 2016).

The dynactin subunit 1 (*DCTN1*) and kinesin family member 5A (*KIF5A*) genes encode for dynactin and kinesin subunits, involved in retrograde and anterograde axonal transport, respectively (Hirokawa et al., 2009; Kwinter et al., 2009). *DCTN1* and *KIF5A* variants have been variably described in ALS (Munch et al., 2004, 2005; Liu et al., 2014, 2017; Brenner et al., 2018; Nicolas et al., 2018), CMT, and dHMN (Crimella et al., 2012; Lopez et al., 2015).

Finally, pleckstrin homology and RhoGEF domain containing G5 (*PLEKHG5*) and mitofusin 2 (*MFN2*) genes, whose mutations are known to cause CMT (Muglia et al., 2001; Zuchner et al., 2004a, 2006; Engelfried et al., 2006; Del Bo et al., 2008; Nicholson et al., 2008; Azzedine et al., 2013; Kim et al., 2013; Iapadre et al., 2018), have been also reported in patients with ALS or an “ALS-like” phenotype (Marchesi et al., 2011; Ozoguz et al., 2015).

## Pathogenic Mechanisms of PNS Damage in ALS

### Peripheral Motor and Sensory Dysfunction in ALS Models

The strongest evidence supporting the dying-back pattern of degeneration comes from transgenic mice overexpressing the mutated form of human SOD1 G93A (hSOD-1^G93A^), the most studied ALS model. Axonal pathology precedes spinal MN death and symptom onset, with the NMJ being the first site of morphological alteration (Fischer et al., 2004). Along with disease progression, the burden of axonal pathology overcomes the moderate MN loss in spinal cord, and suggesting that the motor phenotype in these mice models is mainly driven by the PNS damage (Gould et al., 2006; Hegedus et al., 2007). Indeed, interventions leading to complete rescue of MNs in the spinal cord of mutant SOD1 (mSOD1) ALS mice were not sufficient to halt axonal loss and motor phenotype, suggesting that mechanisms of central, and peripheral degeneration may be at least partially independent (Gould et al., 2006; Rouaux et al., 2007; Suzuki et al., 2007).

Despite the wealth amount of data generated from this model about MN degeneration, important concerns remain about the reproducibility of these findings in human ALS. The preferential involvement of spinal compared to cortical MNs, the anatomical differences in CNS organization between mice and humans and the confounding effects of artificial manipulation, such as transgenic overexpression, limit the translation of ALS preclinical research on patients (Kato, 2008; Philips and Rothstein, 2015). Nonetheless, some studies reported evidence of early NMJ alterations and axonal injury preceding MN loss also in ALS patients (Bradley et al., 1983; Dengler et al., 1990; Fischer et al., 2004; Bruneteau et al., 2015). Moreover, progressive NMJ and motor axon loss is a consistent finding also in other transgenic ALS mice with mutations in *TARDBP*, *FUS*, and *C9orf72* repeat expansion (Picher-Martel et al., 2016). Overexpression of hTDP-43^A315T^ and hTDP-43^WT^ leads to NMJ denervation and loss of corticospinal axons, which in some cases predominate over MN al loss (Wegorzewska et al., 2009; Arnold et al., 2013; Herdewyn et al., 2014). In the hFUS^P525L^ mouse lines, where the mutation is conditionally expressed in MNs, the progressive degeneration is preceded by early pre-symptomatic retraction of motor axons (Sharma et al., 2016).

Reflecting evidence of sensory dysfunction in human ALS, transgenic hSOD-1^G93A^ mice also display neurodegeneration in sensory axon, DRG and proprioceptive sensory fibers of muscle spindles. Signs of axonal damage are detected since the pre-symptomatic stage, progressing with a distal-to-proximal gradient (Guo et al., 2009; Vaughan et al., 2015). Furthermore, loss of dorsal root axons has been detected in rodent models overexpressing mutant TDP-43 and FUS (Huang et al., 2011; Arnold et al., 2013), suggesting that ALS-specific proteins may also affect sensory neurons. However, it is still unclear whether the damage comes from nearby disease spreading or arise independently from MNs. Interestingly, transfection of mutant TDP-43 and SOD1 in cultured sensory neurons exert a negative impact on neurites length and arborization after prolonged culture, suggesting a direct effect (Vaughan et al., 2018).

Therefore, the reported evidence demonstrates that all components of the PNS are affected in ALS, although the differing kinetics of damage and progression still point to differences in vulnerability between the sensory and motor axons and neurons.

### Unraveling Mechanisms of Vulnerability and Resistance of Peripheral Motor and Sensory Neurons in ALS

Studies addressing the mechanisms of PNS involvement in ALS may provide a unique opportunity for unraveling the determinants of the different patterns of vulnerability of motor and sensory neurons. This would allow the identification of specific protective or deleterious factors amenable of therapeutic intervention. Hereafter, we discuss key factors at the PNS level potentially implied in such differences.

#### Intrinsic Axonal Vulnerability

Hyperexcitability of the axolemma, due to altered Na^+^ and K^+^ conductance properties, has been demonstrated in motor but not sensory axons of ALS patients (Shibuya et al., 2011; Park et al., 2017; Matamala et al., 2018). The consequent increase in Ca^2+^ influx may be selectively harmful in the motor compartment, since motor axons show a low Ca^2+^-buffering capacity (Jaiswal, 2013; Leal and Gomes, 2015). In turn, Ca^2+^ overload leads to the activation of effector proteins such as calpain, a calcium-dependent protease, involved in TDP-43 fragmentation, which predisposes to aggregation, Wallerian degeneration and NMJ disassembly (Yamashita et al., 2012; Conforti et al., 2014; Campanari et al., 2016). Specific demise of MNs may also be driven by the involvement of ALS-key proteins, such as TDP-43 and FUS. They are mRNA-binding proteins, mainly localized in the nucleus, which participate in the metabolism of broad pools of mRNAs, by affecting splicing, stability, transport. Mutations in these proteins affect neurons by both loss and gain of function, leading to altered RNA processing, increasing cytoplasmic translocation and propensity to aggregation (Ratti and Buratti, 2016). They are essential during embryonic development, but only MNs in the cortex and anterior horns display sustained expression of both proteins during lifetime (Huang et al., 2010). Although most of the transcriptome is shared between motor and DRG axons, more than one third is unique (Rotem et al., 2017). It has been estimated that TDP43 binds roughly 30% of the whole transcriptome (Ling et al., 2013), including coding and non-coding RNAs, such as miRNA (Kawahara and Mieda-Sato, 2012). TDP-43 is actively recruited in RNA granules along motor axons for active transport (Fallini et al., 2012). Examples of TDP43-bound mRNAs include human neurofilaments, components of the endosomal trafficking and mitochondrial proteins (Stalekar et al., 2015; Schwenk et al., 2016; Wang et al., 2016). Over-expression of wild type and mutant TDP-43 alters the dynamics of its axonal transport (Alami et al., 2014), leading to mitochondrial toxicity and NMJ loss (Lin et al., 2011; Tripathi et al., 2014; Wang et al., 2016). Furthermore, a transient increase in TDP-43 localization along axonal routes is observed following axotomy, suggesting a role in peripheral nerve regeneration (Moisse et al., 2009). However, TDP-43^G348C^ mice show abnormal persistence of cytoplasmic levels after peripheral injury, impairing axonal regeneration (Swarup et al., 2012). Such finding may provide a potential additional explanation to the paucity of regenerative clusters observed in ventral roots and motor nerves of human and animal ALS (Riva et al., 2011b, 2014; Luigetti et al., 2012). In contrast, sensory nerves still show some level of regeneration capacity in ALS patients, suggesting that efficient repair mechanisms may dampen progressive neurodegeneration (Hammad et al., 2007; Isaacs et al., 2007).

#### Neuromuscular Junction

The early susceptibility of the NMJ in ALS relies on peculiar features that distinguish it from other peripheral ending structures, including the sensory receptors. It has high energy demands and requires efficient mechanisms of plasticity and repair to cope with the challenges faced by muscle contraction. Oxidative stress, which contributes to ALS pathogenesis (D’Amico et al., 2013), has a great influence on NMJ function, as the early production of reactive oxygen species (ROS) in distal muscles inhibits transmitter release (Kraft et al., 2007; Naumenko et al., 2011) and leads to synaptic loss (Fischer et al., 2011, Fischer et al., 2012). High levels of oxidative stress have also been observed in muscles and spinal cord of ALS patients (D’Amico et al., 2013; Lanfranconi et al., 2017). Mitochondrial dysfunction further participates to NMJ dismantling, as its role in energy production and Ca^2+^ buffering is essential for proper function and maintenance of the synapse (Fischer-Hayes et al., 2013). Non-neuronal factors are also involved, as terminal schwann cells (TSCs) exert important functions in NMJ stability and repair, adapting their state according to local synaptic environment. Such decoding ability depends on the muscarinic acetylcholine receptor (mAchR), a G-protein-coupled receptor (GPCR) present on the membrane of TSCs, which detects the level of Ach released in the synaptic cleft. SOD1 mutants have been shown to alter the mAchR sensitivity of these cells to local damage, leading to deficient repair of denervated NMJ in ALS mice models (Arbour et al., 2015). Still, such findings need confirmation in humans and other models of ALS, although a relationship between TDP-43 function and TSC-related receptors has been suggested (Arbour et al., 2017).

#### PNS-Related Non-cell Autonomous Toxicity

Besides TSCs, other non-cell autonomous mechanisms in the PNS may further influence MNs vulnerability. The role of Schwann cells in the peripheral nerve has also been recently re-evaluated in ALS. Early deficits in axonal transport may be initially compensated by the transfer of polyribosomes from Schwann cells to the axonal compartment, boosting local protein synthesis as an adaptive response to mSOD1-induced injury (Verheijen et al., 2014). Moreover, Schwann cells may exert partial protection against the increased production of ROS related to axonal degeneration, as the loss of dismutase activity in these cells accelerated disease progression (Lobsiger et al., 2009). However, pathogenic gain-of-functions SOD1 mutants may affect Schwann cells functioning and protective effects as well (Wang et al., 2012). Neuroinflammatory response within the PNS and alterations of the blood-nerve barrier might offer additional insights in the pathogenesis of ALS (Kang and Rivest, 2007; Beers et al., 2008), as suggested by observations raised in mice models (Pupillo et al., 2014; Nardo et al., 2016, 2018) and in human ALS (Devigili et al., 2011; Riva et al., 2011a; Gerevini et al., 2016). Pathological studies of sural nerve biopsies of ALS patients have demonstrated, in a subset of cases, the presence of vasculitic-like inflammatory infiltrates associated with normal nerve morphology, suggesting a potential neuroprotective effect of neuroinflammation within the PNS (Devigili et al., 2011). Indeed, the recruitment of macrophages and stimulation of their phagocytic function in peripheral nerves is important to create a favorable milieu for axonal regeneration, as shown in mSOD1 ALS mice (Nardo et al., 2016).

### Common Grounds for ALS and Hereditary Neuropathies

Despite the distinctive prognosis and disease course, ALS, related MNDs and hereditary neuropathies show a non-negligible degree of overlap in terms of both clinical presentations and genetics. Therefore, a better understanding of the key cellular pathways involved in both diseases may allow a broader comprehension of the molecular events leading to axonal and MN degeneration in ALS.

#### Neuronal Cytoskeleton

Microtubules, NFs and actin are the main components of axonal cytoskeleton, and impairment in either structure or functioning of any of these components may lead to axonal atrophy, retraction and degeneration or transport defects (Gentil et al., 2015; Kevenaar and Hoogenraad, 2015). Elevated levels of light NFs (NFL) and phosphorylated heavy NFs have been observed in the cerebrospinal fluid and serum of ALS, together with lower mRNA NFL levels in MNs (Volkening et al., 2009; Gaiani et al., 2017; Feneberg et al., 2018). Accumulation of NF components in axons is a prominent feature of both human and animal ALS (Leigh and Swash, 1991; Rouleau et al., 1996; Morrison et al., 2000), and has been observed along both motor and sensory axons in the hSOD-1^G93A^ ALS mouse model (King et al., 2012; Gentil et al., 2015; Sassone et al., 2016). Alterations of ALS-key genes, such as *SOD1* and *TARDBP*, have been shown to affect the stability of NFL mRNA, suggesting that altered stoichiometry between the different NF components may contribute to the disease (Volkening et al., 2009). Mutations in *NEFL* and *NEFH*, leading to axonal CMT, promote protein aggregation, aberrant mitochondrial morphology and shortening of axonal length (Yoshihara et al., 2002; Rebelo et al., 2016; Nam et al., 2017). Tubulin Alpha 4a (*TUBA4A)*, encoding for α-tubulin, has been recently identified as a novel ALS gene (Smith et al., 2014). It has been shown that *TUBA4A* mutations are able to alter microtubule dynamics, with depolymerization and degradation of α-tubulin (Helferich et al., 2018). Although this gene has been described only in cohorts of ALS and FTD patients, a sensory and motor neuronopathy is observed in progressive motor neuronopathy mice mutated in tubulin binding cofactor E (*TBCE*), a known interactor of α-tubulin (Schafer et al., 2017). Profilin 1 (*PFN1*) has been found as a rare cause of familial ALS (Wu et al., 2012). It is a crucial protein for the conversion of monomeric (G)-actin to filamentous (F)-actin. Interestingly, although pathogenic variants do not disrupt actin dynamics (Freischmidt et al., 2015), alterations in microtubule growth and increased propensity to cytoplasmic TDP43 aggregation have been described (Matsukawa et al., 2016; Tanaka et al., 2016; Henty-Ridilla et al., 2017), revealing potential cell-type specific mechanisms of neurodegeneration.

#### Axonal Transport

Defects in axonal transport have been clearly observed both in ALS and dHMN. SOD1 and TDP43 mutants impair anterograde and retrograde axonal transport, leading to loss of essential synaptic components, mitochondrial abnormalities, and neurite shortening (Williamson and Cleveland, 1999; Perlson et al., 2009; Alami et al., 2014). Similarly abnormal transport of organelles, such as mitochondria and the endoplasmic reticulum (ER), is found in CMT models due to *NEFL* mutations (Tradewell et al., 2009). The failure of axon-cell body communication may dampen the activation of repair mechanisms, thus conferring an increase vulnerability of the peripheral compartment, more pronounced for the distal segments (Fischer et al., 2004; Dadon-Nachum et al., 2011). Strategies aiming at reinforcing axonal transport may thus confer protection to a broad range of pathogenic alterations, preserving the axonal routes and its endings.

#### Protein Folding

The highly differentiated and post-mitotic state of MNs exerts a relevant pressure over the protein quality control system of the cell, which is devoted to the correct folding, function and turnover of the whole proteome. HSPB1/27, HSPB8/22, and sigma non-opioid intracellular receptor 1 (SIGMAR1) are three chaperones with a significant expression levels in MNs (Mavlyutov et al., 2010). Mutations of the small heat shock proteins have been mostly associated with a dHMN or axonal CMT2 phenotype, while *SIGMAR1* gene variants have been reported also in rare ALS patients (Al-Saif et al., 2011; Rossor et al., 2012). HSPB1 mutants alter both its chaperone activity in NF organization and preservation of axonal stability (Evgrafov et al., 2004; Ackerley et al., 2006). HSPB8 mutant mice develop a progressive motor neuropathy through specific neurite degeneration (Irobi et al., 2010; Bouhy et al., 2018). In addition, alterations in TDP-43 expression, and associated splicing is observed in the muscles of these mice (Cortese et al., 2018). Conversely, there is a significantly higher HSPB8 expression in surviving neurons in the hSOD-1^G93A^ mouse model of ALS (Crippa et al., 2010). Finally, SIGMAR1, together with MFN2 and vesicle-associated membrane protein-associated protein B/C (VAPB), are able to modulate unfolded protein response (UPR) sensors, including protein kinase RNA-like endoplasmic reticulum kinase (PERK) (Nguyen et al., 2015).

#### Mitochondrial/Associated Membranes (MAMs)

The ER and mitochondria have evolved complex sites of interactions, defined as mitochondrial/associated membranes (MAMs), which are important for essential cellular functions, such as calcium homeostasis, autophagy, regulation of mitochondrial dynamics, and axon survival (Giorgi et al., 2015). Both ALS and hereditary neuropathies display dysfunction in one or more of MAM components. A huge number of cellular proteins participate in MAMs, including MFN2 and VAPB, which act as tethering sites between the two organelles (Bernard-Marissal et al., 2015). VAPB mutants affect the interaction with the mitochondrial protein tyrosine phosphatase-interacting protein 51 (PTPIP5) (de Brito and Scorrano, 2008; De Vos et al., 2012). Interestingly, TDP43 and FUS mutants have been shown to decrease ER-mitochondria contacts by disrupting VAPB-PTPIP5 binding (Stoica et al., 2014). MFN2 mutants alter other MAM functions, such as mitochondrial transport, fusion, and autophagosome assembly (Kim et al., 2015). Loss-of function mutations in *SIGMAR1* display decreased ER-mitochondria association, resulting in MN death, and axonal degeneration due to impaired retrograde transport (Bernard-Marissal et al., 2015; Watanabe et al., 2016).

## Therapeutic Perspectives

Due to the high similarity between these neuromuscular disorders and the intimate relationship observed in the molecular pathways involved, it may not be surprising if functional proteins involved in either of the two diseases may provide benefits on their respective counterpart.

Preservation of NMJ is an attractive target to delay muscle denervation and disability in ALS. A drug-screening platform in *C. elegans* and zebrafish models of ALS identified pimozide, an already approved neuroleptic, as a NMJ stabilizer by blocking T-type Ca^2+^ channels (Patten et al., 2017). Despite some benefits in neurophysiologic measures were reported in hSOD-1^G93A^ mice and human patients in the first report, one study assessing long-term effects showed warned caution, as worsening of survival and muscle function were observed in mutant SOD1, and TDP-43 mice models (Pozzi et al., 2018). Notably, an agonist antibody directed toward muscle-specific kinase (MuSK), a post-synaptic tyrosine kinase receptor essential for NMJ maintenance, preserved motor synapses, delayed muscle denervation and extended lifespan in hSOD-1^G93A^ ALS mice (Cantor et al., 2018). The promising findings of this study still awaits confirmation and replication in other disease models.

Reinforcing the action of factors involved in axonal stability and transport may restore soma-axon communication, delaying NMJ loss and axonal degeneration. Pharmacological inhibition of histone deacetylase 6 (HDAC6), a known client protein of HSPB1, was able to increase the stability of microtubules, rescuing axonal loss, and improving outcome in a CMT2F mouse model with mutant HSPB1 (d’Ydewalle et al., 2011). Interestingly, HDAC6 inhibition was also able to restore axonal transport defects in patient-derived MNs mutated in FUS (Guo et al., 2017). Pharmacologic agents targeting microtubule dynamics, such as noscapine and vinblastine, delayed disease onset and prolonged survival in hSOD-1^G93A^ mice, by reducing microtubule turnover (Fanara et al., 2007).

Inhibition of p38/mitogen-associated protein kinase (MAPK) signaling in MNs was able to restore retrograde axonal transport defects *in vivo* (Gibbs et al., 2018), together with reduced microglia-induced neuroinflammation in the spinal cord (Kim and Choi, 2010; Zhan et al., 2015; Leal-Lasarte et al., 2017). However, it is remarkable that no significant improvement was observed on the clinical phenotype (Gibbs et al., 2018), suggesting that inhibition of this pathway alone is not sufficient to fully rescue MN function.

Reducing ER stress through either a potentiation of the chaperone system or inhibition of stress-related factors may limit the production rate of misfolded proteins. Enhancing HSPB8 activity has been shown to be protective against accumulation of TDP43 aggregates in MNs (Crippa et al., 2016; Rusmini et al., 2017), and to extend survival of hSOD-1^G93A^ mice (Aurelian et al., 2012). Trehalose, a chemical chaperone known to induce HSPB8, reduces ER stress levels and improve autophagy, delaying disease, and prolonging MN survival in a mouse model (Zhang et al., 2014; Li Y. et al., 2015). Upon UPR activation, cells switch to a state of translational repression and stimulation of chaperones’ synthesis, processes regulated by phosphorylation of the eukaryotic translation initiation factor 2A (eiF2α). Salubrinal and guanabenz, two eiF2α phosphatase inhibitors, protected against MN degeneration in ALS mice (Saxena et al., 2009;Wang et al., 2014), although one study reported that guanabenz accelerated disease progression in an ALS model (Vieira et al., 2015).

Considering their importance in cell physiology, MAMs could represent an important target for therapy in ALS and may prove beneficial for a wide array of downstream signaling cascades relying upon these structures. An agonist of SIGMAR1 was shown to improve muscle activity and motor performance in pre-symptomatic hSOD-1^G93A^ ALS mice (Mancuso et al., 2012). Notably, overexpression of MFN2 prevented NMJ loss and delayed onset and progression in a murine model by raising calpastatin levels, a calpain inhibitor, essential for survival, and function of the NMJ (Wang et al., 2018).

Boosting axonal regeneration through either neuron- or Schwann-targeting factors may also be a valuable alternative strategy in order to preserve motor function (Gilley et al., 2017). Studies modulating Neuregulin 1 activity, a neurotrophic factor involved in peripheral nerve development and regeneration, in murine hSOD-1^G93A^ ALS lead to increased re-innervation and MN survival (Mancuso et al., 2016; Modol-Caballero et al., 2017). However, other approaches aimed at slowing Wallerian degeneration, such as the mutations in the Slowed Wallerian degeneration (Wld^S^) and Sterile Alpha and TIR Motif Containing 1 (Sarm1) proteins, gave only modest or no results in terms of progression and survival (Fischer et al., 2005; Peters et al., 2018), suggesting that alternative mechanisms may underlie the axonal loss observed in ALS.

## Conclusion

Clinical and pathological studies in both human patients and rodent models suggest that the PNS is involved in ALS pathogenetic cascade. A better comprehension of the molecular events occurring into the PNS may prove essential for a better understanding of MND pathomechanisms and may pave the way to the discovery of novel therapeutic targets. Considering that in ALS neurodegeneration derives from the combination of several dysfunctional pathways, it might be possible that the development of combination therapies acting on multiple cascades located in both the central and the peripheral compartments may show synergistic neuroprotective effect in order to prevent axonal degeneration, MN cell death, und ultimately in prolonging patients’ survival.

## Author Contributions

FG and SS contributed to the collection of available evidence and writing. YF, AQ, NR, CL, and LT contributed to the manuscript writing and review of intellectual content of the manuscript, and approved the final version of the manuscript. AQ and NR contributed to the manuscript design.

## Conflict of Interest Statement

The authors declare that the research was conducted in the absence of any commercial or financial relationships that could be construed as a potential conflict of interest.
